# Dimerization and Heme Binding Are Conserved in Amphibian and Starfish Homologues of the microRNA Processing Protein DGCR8

**DOI:** 10.1371/journal.pone.0039688

**Published:** 2012-07-02

**Authors:** Rachel Senturia, Arthur Laganowsky, Ian Barr, Brooke D. Scheidemantle, Feng Guo

**Affiliations:** 1 Department of Biological Chemistry, David Geffen School of Medicine, Molecular Biology Institute, University of California Los Angeles, Los Angeles, California, United States of America; 2 Department of Chemistry and Biochemistry, UCLA-DOE Institute for Genomics and Proteomics, University of California Los Angeles, Los Angeles, California, United States of America; University of South Florida College of Medicine, United States of America

## Abstract

Human DiGeorge Critical Region 8 (DGCR8) is an essential microRNA (miRNA) processing factor that is activated via direct interaction with Fe(III) heme. In order for DGCR8 to bind heme, it must dimerize using a dimerization domain embedded within its heme-binding domain (HBD). We previously reported a crystal structure of the dimerization domain from human DGCR8, which demonstrated how dimerization results in the formation of a surface important for association with heme. Here, in an attempt to crystallize the HBD, we search for DGCR8 homologues and show that DGCR8 from *Patiria miniata* (bat star) also binds heme. The extinction coefficients (*ε*) of DGCR8-heme complexes are determined; these values are useful for biochemical analyses and allow us to estimate the heme occupancy of DGCR8 proteins. Additionally, we present the crystal structure of the *Xenopus laevis* dimerization domain. The structure is very similar to that of human DGCR8. Our results indicate that dimerization and heme binding are evolutionarily conserved properties of DGCR8 homologues not only in vertebrates, but also in at least some invertebrates.

## Introduction

microRNAs (miRNAs) are a class of ∼22 nucleotide (nt) non-coding RNAs that negatively regulate gene expression by destabilizing target mRNAs or inhibiting their translation [1], [2]. Mature miRNAs originate from primary transcripts (pri-miRNAs) that may be transcribed as introns of mRNAs or as independent transcripts [3]. In the first step of miRNA processing, a pri-miRNA is cleaved in the nucleus to produce an intermediate called the precursor miRNA (pre-miRNA), by a protein complex called the Microprocessor, which is minimally composed of the ribonuclease III enzyme Drosha, and the RNA-binding partner DGCR8 [4], [5], [6], [7], [8]. pre-miRNAs are exported to the cytoplasm where they undergo additional cleavages by another ribonuclease III enzyme Dicer to produce miRNA duplexes. miRNA duplexes are then incorporated into the miRNA-induced silencing complex (miRISC) and unwound into the mature single-stranded form. DGCR8, the focus of this study, is required for pri-miRNA processing both *in vitro* and *in vivo* [6], [8], [9], [10]. The DGCR8 gene is heterozygously deleted along with about 30 other genes in DiGeorge syndrome patients [11]. Dgcr8^+/−^ mouse models indicated pri-miRNA processing defects in the brain and neurological defects and symptoms similar to those observed in DiGeorge syndrome [12], [13], [14].

**Figure 1:**
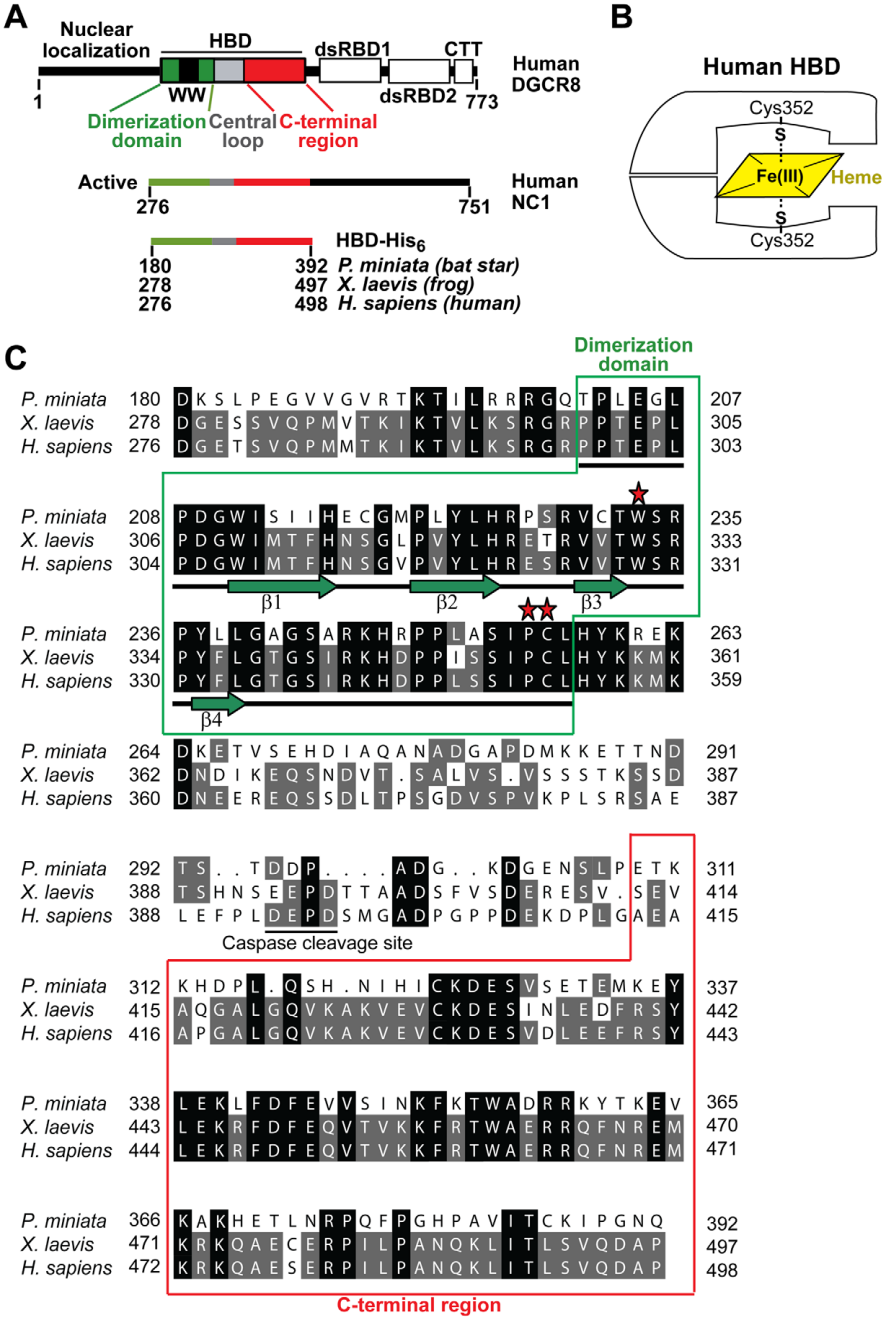
Domain structure of DGCR8 and sequence alignment of the heme-binding domains. (A) Domain structure of human DGCR8 and schematics of the NC1 and HBD constructs used in this study. (B) Schematic of how the DGCR8 HBD binds Fe(III) heme. (C) Sequence alignment of bat star, frog and human HBDs. Identical residues are shaded in black. Residues that are identical only between two species are shaded in gray. Red stars denote residues in human HBD known to be important for heme binding. Secondary structure assignments derived from the crystal structure of frog dimerization domain are shown below the sequences, with β-strands as green arrows and loops as bars.

In addition to the well accepted role of recognizing pri-miRNAs [15], [16], [17], [18], we found that a truncated form of human DGCR8 called NC1 (residues 276–751) binds Fe(III) heme when overexpressed in *E. coli* and is fully active in reconstituted pri-miRNA processing assays [16], [19]. ApoNC1 dimer is activated for pri-miRNA processing *in vitro* by Fe(III) (ferric) heme, but not by Fe(II) (ferrous) heme [20]. These observations demonstrated the functional importance of heme binding to DGCR8, especially the redox state of the heme iron, in miRNA processing. Human DGCR8 is composed of an N-terminal region that is required for nuclear localization, a central heme-binding domain (HBD, residues 276–498), two double-stranded RNA-binding domains (dsRBDs, residues 500–700) and a C-terminal tail (CTT, residues 701–751) (Figure 1A). Recombinant human HBD binds Fe(III) heme and displays an electronic absorption spectrum similar to that of Fe(III) heme-bound NC1 [21]. The HBD is a dimer and each HBD dimer binds one heme molecule. The HBD coordinates the heme Fe(III) using two thiol/thiolate groups from the highly-conserved Cys352 residues contributed by both subunits (Figure 1B) [19].

We previously identified a conserved N-terminal region of human HBD as a dimerization (sub)domain (residues 276–353), and determined a crystal structure of the human DGCR8 dimerization domain [21]. This structure revealed an expansive dimerization interface mediated mainly by hydrophobic interactions. The structure contains a WW motif, which is comprised of three anti-parallel β-strands [21]. Most previously characterized WW motifs are monomeric and mediate protein-protein interactions by associating with proline-rich peptide segments [22], [23]. The WW motif of DGCR8 is structurally similar to other known WW motifs [21]. However, the WW motif in DGCR8 is unlikely to bind proline-containing peptides because the surface typically used for peptide binding is occluded in the structure. Instead, the DGCR8 WW motif forms a structural platform for dimerization and aids heme binding [21]. Dimerization occurs in part through formation of a β-sheet between the WW motif and a fourth strand in the C-terminal neighboring region (residues 332–352) of the partner subunit. This “domain swapped” conformation allows proper spatial positioning of residues required for heme binding, thus resulting in a heme-binding surface [21]. The structure of the human DGCR8 dimerization domain provides structural evidence to explain why dimerization of DGCR8 is required for heme binding.

Our previous studies have been mostly focusing on the human DGCR8 protein. Here we explore heme binding properties of DGCR8 homologues, partially in pursuit to crystallize the HBD. We show that the bat star (*P. miniata*) DGCR8 binds Fe(III) heme. We present the crystal structure of the frog (*X. laevis*) DGCR8 dimerization domain. These results suggest that dimerization and heme binding are evolutionarily conserved features of the DGCR8 family of miRNA processing factors.

## Results

### Heme Binding is Conserved in a Starfish DGCR8

In our pursuit to crystallize the DGCR8 HBD, we searched for homologues within other organisms. DGCR8, along with its partner nuclease Drosha, was only found in animals. We noticed that while the sequences of mammalian DGCR8 HBDs are highly similar to each other (for example, the mouse sequence is 96% identical to the human), those from frog (*Xenopus laevis*) and bat star (*Patiria miniata*), an echinoderm invertebrate [24], have diverged from human (Figure 1C). The bat star HBD sequence shares lower identity (41%) to human than that of frog HBD (80%). Interestingly, the residues known to be critical for heme binding in human, Cys352 [16], Trp329 [21] (Figure 1C, red stars), are conserved in all three species. These observations suggest that the bat star and frog DGCR8 may use a similar mechanism to bind heme as the human.

**Figure 2:**
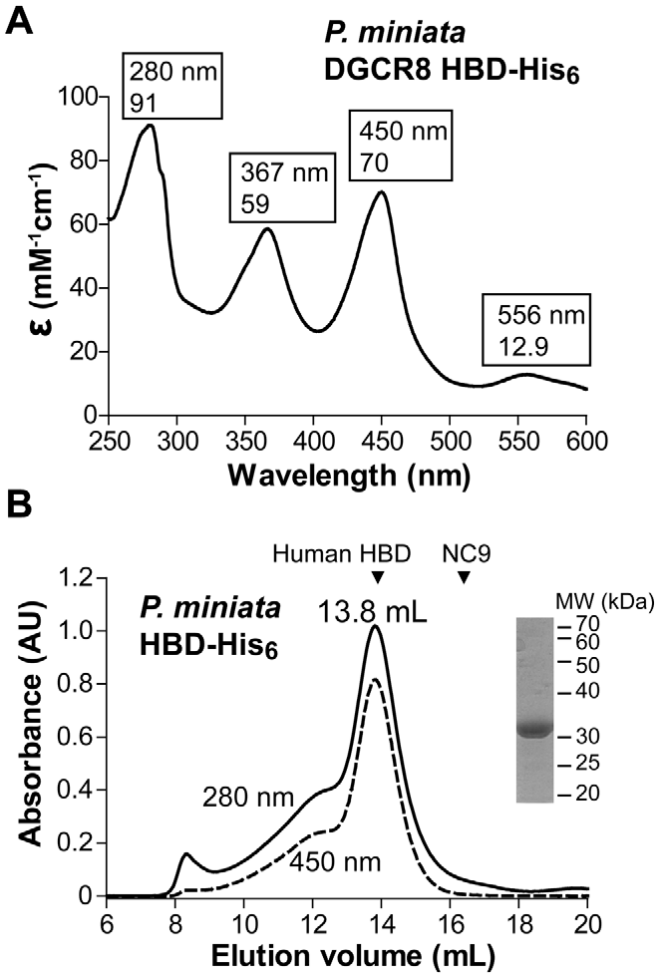
The bat star DGCR8 HBD binds heme as a dimer. (A) Electronic absorption spectrum of bat star HBD. Peak wavelengths and the corresponding extinction coefficients are labeled. (B) Size exclusion chromatogram of the bat star HBD, obtained from the last step of the purification procedure. The elution volumes of the dimeric human HBD (54 kDa) and the monomeric human NC9 (29 kDa) proteins are indicated as triangles. *Inset*, a sodium dodecyl sulfate polyacrylamide gel electrophoresis (SDS-PAGE) image of the 13.8-mL peak fraction of bat star HBD.

To experimentally test heme binding and to seek new opportunities for crystallization, we cloned, expressed in *E*. *coli*, and purified the bat star and frog HBD-His_6_ (all HBD constructs this paper refers to contain a non-cleavable C-terminal His_6_ tag; they are abbreviated as HBD). The frog HBD has been shown to bind Fe(III) heme [19]. Here we report that, the purified bat star HBD displays an electronic absorption spectrum with peaks at 367, 450 and 556 nm, similar to those of human HBD, indicating that it also binds Fe(III) heme (Figure 2A). In size exclusion chromatography (SEC) analyses, the bat star HBD (26 kDa per subunit) eluted at 13.8 mL, which is similar to that of the human HBD dimer (54 kDa) but is 2.6 mL earlier than that of a monomeric human DGCR8 protein (residues 499–751, called NC9, 29 kDa). This observation indicates that the Fe(III) bat star HBD is a dimer (Figure 2B). These results suggest that heme binding and dimerization are conserved at least among DGCR8 homologues from mammals, amphibians and starfish.

### Determination of the Extinction Coefficients and Heme Contents of Fe(III) Heme-DGCR8 Complexes

To increase the chance of obtaining crystals and to facilitate biochemical analysis of heme-DGCR8 complexes, we need to estimate the heme occupancy of purified complexes. Overexpression of heme proteins in bacteria induces heme deficiency and often results in the presence of both holo- and apo-proteins. Even though we add δ-aminolevulinic acid (δ-ALA), a heme biosynthesis intermediate and the product of the rating-limiting step in heme biosynthesis pathway, the *A*_450 nm_/*A*_280 nm_ ratio (the *RZ* value) of recombinant DGCR8 proteins as calculated from electronic absorption spectra often varies from preparation to preparation. Until the current study, it has not been clear to what extent these proteins were occupied by heme.

To answer this question, we determined the extinction coefficients of various DGCR8-heme complexes, which in turn allowed us to estimate their heme occupancy using electronic absorption spectroscopy. For each DGCR8-heme complex, we recorded the electronic absorption spectrum, determined the heme concentration using the pyridine hemochromogen method [25], and measured the protein concentration using the Bicinchoninic Acid (BCA) protein assay [26]. The pyridine hemochrome, formed after extraction of heme from the complexes and reduction of the heme iron, has a sharp and intense absorption peak at 557 nm that obeys Beer’s law over a wide concentration range, allowing the concentration of heme in the complex to be determined accurately [25]. The heme concentration of a bat star HBD preparation was determined to be 7.3 µM, and the extinction coefficient of the Fe(III) heme in this protein at 450 nm (ε_450,heme_) was determined to be 70 mM^−1^ cm^−1^ (Figure 2A and Table 1). The BCA method measures cuprous (Cu^1+^) ion produced in the reaction of proteins with alkaline Cu^2+^ (called the biuret reaction) and has relatively low protein-to-protein variation [26]. The concentration of the same bat star HBD dimer protein described above was estimated to be 7 µM. Thus the molar ratio of protein to heme, or heme occupancy, was close to 100%. With the confidence in the homogeneity of this complex, we further estimated the extinction coefficient of the dimeric Fe(III) heme-bound HBD at 280 nm (ε_280,holo_) to be 91 mM^−1^cm^−1^ (Figure 2A). This value is higher than that of the dimeric apoHBD (ε_280,apo_  = 48 mM^−1^cm^−1^) as calculated from the amino acid sequence [27], consistent with the expectation that the extinction coefficient of the HBD complex at 280 nm is contributed by both the protein and heme moieties. We encounter situations where apoHBD is present in the bat star HBD samples and the *A*_450_/*A*_280_ ratio is lower than ε_450_/ε_280,holo_ (0.77). In these cases, the heme occupancy (*O*_heme_) may be calculated using the following equation:
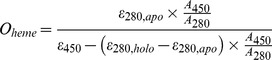



**Table 1:** Extinction coefficients for homologous HBDs.

	ε_366/367_ (mM^−1 ^cm^−1^)	ε_450/451_ (mM^−1 ^cm^−1^)	ε_556_ (mM^−1 ^cm^−1^)
**Human HBD**	60±1 (366 nm)	74±1 (450 nm)	14.2±0.3
**Frog HBD**	62±3 (366 nm)	72±3 (451 nm)	14.0±0.6
**Bat star HBD**	59±3 (367 nm)	70±4 (450 nm)	12.9±0.6
**Human NC1**	62±2 (366 nm)	74±2 (450 nm)	14.1±0.4

Using the pyridine hemochromogen method, we determined the ε_450_ values of the human HBD and NC1 proteins and the ε_451_ of the frog HBD to be 74, 74 and 72 mM^−1^cm^−1^, respectively (Table 1). The essentially identical ε_450/451_ values suggest that the chemical environments of the Fe(III) heme are very similar in the HBDs from human, frog and bat star, and regardless whether the dsRBDs and CTT are present. The ε_450_ of the human NC1 is higher than the value we previously reported (58 mM^−1^cm^−1^) [16]. However, the latter was determined via organic solvent extraction of heme, followed by quantification using reverse-phase high pressure liquid chromatography (HPLC). This procedure may have underestimated the extinction coefficient value if the heme extraction was incomplete.

The BCA assays estimated that the heme occupancy of the human HBD, NC1 and frog HBD preparations used in these experiments were less than 100%, thus their ε_280,holo_ could not yet be confidently determined. We note that the absence of imidazole and thiol-containing reducing reagents in the storage buffer of bat star HBD may have contributed to the higher heme content. Further investigation will be needed to confirm this possibility.

### Structure Determination of the Frog DGCR8 Dimerization Domain

**Figure 3:**
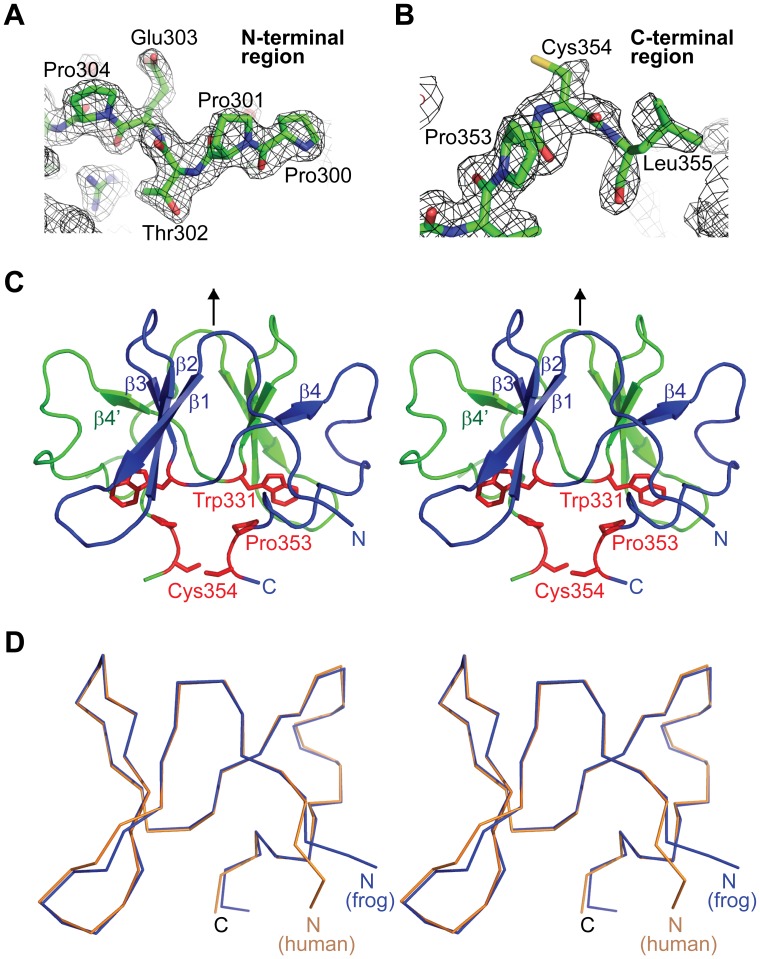
Crystal structure of the frog DGCR8 dimerization domain. (A–B) 2F_o_-F_c_ electron density maps, contoured at 1σ level, of the N- and C-terminal regions of the frog dimerization domain, respectively. (C) Wall-eyed stereo diagram of the crystal structure of frog dimerization domain. The dimer subunits are colored green and blue. Secondary structures from the green subunit are denoted with a prime. The crystallographic two-fold axis relating the two subunits is indicated by the arrow. Residues known to be important for heme binding are highlighted in red. (D) Superimposition of human (orange) and frog (blue) dimerization domain Cα traces shown in stereo.

In initial crystallization screens, the frog HBD protein appeared as red phase separation. An additive screen was performed under the condition producing this phase separation. Single crystals were obtained from the addition of tribasic sodium citrate. These crystals lacked any color, suggesting that heme was not bound to the protein. Nevertheless, they diffracted X-ray to 1.9 Å resolution and a complete data set was collected (Table 2). The structure was solved using molecular replacement with the human dimerization domain structure as the search model. In the 2F_o_−F_c_ map, clear and continuous electron density was observed from Pro300 to Cys354; the electron densities of the main chain and side chain of Leu355 were present but were disconnected when contoured at 1σ level (the N- and C-terminal ends of the electron density are shown in Figures 3A, 3B). We did not detect electron density for the first 22 N-terminal residues (#278–299) and the 142 residues at the C-terminus (#356–497). The latter region is comprised of the central loop and the C-terminal region of the frog HBD. Crystallographic refinement was performed using a model containing DGCR8 residues 300–355 and water molecules, and resulted in final R and R_free_ factors of 0.2068 and 0.2280, respectively (Table 2).

**Table 2:** Crystallographic statistics of the structure of the frog DGCR8 dimerization domain.

Data collection	Native
Space group	P4_3_2_1_2
Cell dimensions	
a, b, c (Å)	39.89, 39.89, 82.13
α, β, γ (°)	90, 90, 90
Resolution (Å)	80–1.9 (1.97–1.9)
Observed reflections	64,720
Unique reflections	5,640
Wavelength (Å)	1.54
R_sym_	0.1 (0.459)
I/σ(I)	21.23 (3.38)
Completeness (%)	99.1 (99.6)
Redundancy	11.5 (6.6)
**Refinement**	
Resolution (Å)	35.9–1.9
No. of reflections used	5,593
R_work_	0.2068
R_free_	0.2280
Average B factor (Å^ 2^)	
protein/water	34.773/37.255
Root mean square deviation	
bond length (Å)/angles (°)	0.007/1.081
**Content of asymmetric unit**	
No. of protein molecules	1
No. of protein	
Residues/atoms	56/454
No. of solvent atoms	33
**Ramachandran statistics**	
Allowed/generous/disallowed (%)	100/0/0

*R*_sym_ = ∑*_hkl_*∑*_i_* |*I_i_*(*hkl*)−<*I*(*hkl*)> |/∑*_hkl_* ∑*I_i_*(*hkl*). *R*_work_ = ∑|*F_o_*–*F_c_*|/∑*F_o_*. *R*_free_ = ∑|*F_o_*−*F_c_*|/∑*F_o_*, calculated using a random set containing 10% reflections that were not included in refinement.

**Figure 4:**
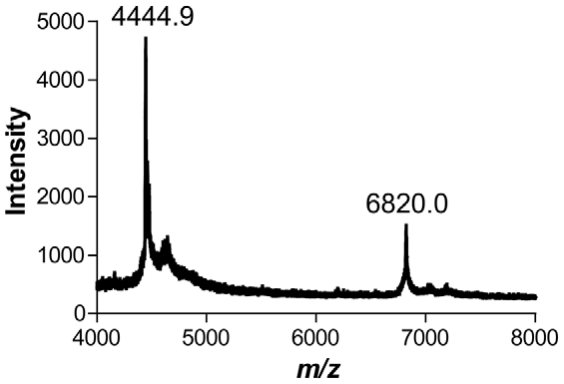
MALDI-TOF mass spectrometry analysis of crystals obtained from frog DGCR8 HBD. Ions with a “+1” charge state are labeled with their corresponding *m/z* values. The ion with *m/z* of 6820.0 roughly corresponds to the dimerization domain observed in the crystal structure.

To determine if the missing electron density was due to lack of ordered structures in the crystal or degradation of the HBD protein over the prolonged period of crystallization, we dissolved crystals and analyzed the protein using matrix-assisted laser desorption ionization time of flight (MALDI-TOF) mass spectrometry. The resulting mass spectra did not reveal any full-length frog HBD at the expected molecular mass of 26,469 Da, as we have successfully achieved for the same protein using the same instrument and a similar procedure in a previous study [19]. Instead, we observed two main ions with *m/z* of 4,445 and 6,820, respectively (Figure 4). These ions correspond to protein segments with molecular masses of 4,444 Da and 6,819 Da. The molecular mass of the residues observed in the continuous electron density of the crystal structure is 6,371 Da, which is close to the mass of the ion observed at *m/z* of 6,820. Inclusion of neighboring Arg299, His356, and Tyr357 into the calculation produces a fragment with a molecular mass of 6,828 Da, which is very close to the mass observed via mass spectrometry. Thus, our crystals contained the dimerization domain generated through degradation of the HBD. Some neighboring residues likely existed in the polypeptide chain in the crystals, but adopted flexible conformation. We have not been able to identify the protein fragment corresponding to the 4,445 *m/z* peak, either in the electron density or via additional biochemical analyses due to the limited amount of material. It is possible that this peak represents a degradation product from the dimerization domain or from another region of the HBD. In the mass spectrum, the intensity of the 4,445 *m/z* peak is higher than that of the 6,820 *m/z* peak (Figure 4). However, because ionization efficiency often negatively correlates with molecular mass, the 4444-Da species is not necessarily more abundant than the 6819-Da species in the crystals.

### Structural Conservation of Dimerization and Heme Binding

The frog dimerization domain crystal contains a single polypeptide chain in the asymmetric unit. The chain forms a dimer via a crystallographic twofold symmetry (Figure 3C). The subunits in each dimer are held together by an extensive interface mainly mediated by hydrophobic interactions. The WW motif folds into three β-strands (β1–β3). After strand β3, the polypeptide chain extends through a hinge loop into a fourth β-strand (β4) that interacts with the WW motif of the partner subunit, forming a continuous β-sheet and resulting in an apparently domain-swapped dimer. All DGCR8 residues known to be important for heme binding, including Pro351 [19], Cys352 [16] and Trp329 [21], cluster on a common surface (shown in red in Figure 3C). With a modest conformational change, this surface could form a pocket to accommodates the Fe(III) heme and allow the two Cys352 side chains to coordinate the heme iron from both sides of the heme plane.

The dimerization domain structure of frog DGCR8 is very similar to that of human DGCR8 with some differences at the N- and C-termini. Superimposition of the Cα atoms of the two structures (Figure 3D) results in a low overall root mean square deviation of 0.38 Å. This is not surprising given that only three residues out of a total of 55 amino acids are different between the human and frog dimerization domain sequences (Figure 1C). Pro300 and Pro301 at the N-terminus of the frog structure are shifted by about 7 Å and 3 Å, respectively (Figure 3D). The human DGCR8 dimerization domain was crystallized in the presence of the N-terminal 22 amino acids (residues 276–297) [21]. Even though these residues are disordered, with no electron density observed, they may have contributed to the structural differences at the N-terminus. In the structural superimposition, the side chain of frog Cys354 points toward a direction distinct from that of human Cys352, as supported by a relatively weak but substantial electron density (Figure 3B). This observation suggests that the conformation of this axial ligand of Fe(III) heme is flexible, due to absence of heme and/or close proximity to the C-terminus. Overall, the high degree of sequence and structural conservation suggests that frog DGCR8 uses the same mechanism of dimerization to aid heme binding as identified in human DGCR8 [19], [21].

## Discussion

Here we explore the heme binding and dimerization properties of DGCR8 homologues. We show that both frog and bat star homologues bind Fe(III) heme in the same manner as human DGCR8. The structure of the dimerization domain of frog DGCR8 is nearly identical to that of human DGCR8.

Among the known domains of DGCR8, the WW motif-containing dimerization domain is a defining feature of the DGCR8 family proteins. The sequences of over 40 DGCR8 homologues are available in the UniProt Knowledgebase (UniProtKB) and National Center for Biotechnology Information (NCBI) databases. All of them can be uniquely identified when only the dimerization domain sequences are used in the search. In contrast, the two dsRBDs arranged in tandem (dsRBD1 and dsRBD2) are found in many RNA-binding proteins that function either in other steps of miRNA biogenesis, such as the HIV trans-activator RNA (TAR)-binding protein (TRBP) and the protein kinase R (PKR)-activating protein (PACT) [28], [29], or in pathways not known to be directly related to miRNA processing (such as Staufen) [30]. Our studies show that both sequence and structure of the dimerization domain are highly conserved ([21] and herein).

The dimerization domain is an integral part of the heme-binding domain in human DGCR8; it aligns residues important for heme-binding in proper spatial positions (Figure 3C) [21]. We are interested to investigate if heme binding is also a conserved feature of DGCR8. Beyond the dimerization domain, the central loop region (containing at least residues 377–410 in human DGCR8) is dispensable for heme binding and is poorly conserved (Figure 1C). We recently showed that a function of the central loop is to present a site for cleavage of DGCR8 by caspases, which results in inhibition of its pri-miRNA processing activity [31]. The C-terminal region of the HBD (residues 411–498 in humans) is required for heme binding [21] and is modestly conserved. It is not clear yet how the C-terminal region contributes to association with heme. Furthermore, DGCR8 is the only known heme protein that uses two cysteine side chains as coaxial ligands for binding Fe(III) heme [19]. The axial ligand Cys352 and its immediate neighboring residue Pro351 are completely conserved. Trp329, which is also important for human DGCR8 to bind Fe(III) heme, is conserved in mammals, birds, lizards, amphibians, fish and starfish, but not in insects and worms. The relationship between conservation of Trp329 and heme binding will be addressed in a separate study. Overall, our study supports the Fe(III) heme-mediated activation of pri-miRNA processing as a common mechanism not only among vertebrates, but also in at least some invertebrates.

## Materials and Methods

### Plasmid Construction

The coding sequence of bat star DGCR8 HBD (residues 180–392), was amplified from a partial cDNA (NCBI Accession number GQ397480) using PCR and was cloned into pET-24a^+^ (Novagen), between the NdeI and XhoI sites. The coding sequence of the plasmid was verified via sequencing.

### Protein Expression and Purification

The human, frog and bat star HBD-His_6_ proteins were expressed in *E. coli* strain BL21-CodonPlus (DE3)-RIPL (Stratagene, a part of Agilent Technologies) and purified using Ni-affinity chromatography followed by size exclusion chromatography, similar to the procedure previously used for human HBD-His_6_ [21]. A heme biosynthesis intermediate δ-aminolevulinic acid (MP Biomedicals) was added to a final concentration of 1 mM at the time of induction. In Ni^2+^ affinity chromatography, both the lysis and wash buffers contained 500 mM NaCl, 10 mM imidazole, and 20 mM Tris pH 8.0; the elution buffer contained the same components except 200 mM imidazole. The SEC buffer contained 400 mM NaCl, 20 mM Tris pH 8.0, and 1 mM dithiothreitol (DTT). In the purification of bat star HBD-His_6_, the protein was immediately buffer-exchanged into 400 mM NaCl and 20 mM Tris pH 8.0 after affinity chromatography, prior to storage at 4°C overnight and the SEC purification step in the following day. The buffer exchange used here and below was achieved using Amicon Ultracel centrifugal concentrators with Molecular Weight Cutoff’s of 10 or 30 kDa (EMD Millipore, Billerica, MA).

The human NC1 protein was expressed and purified as previously described [16].

### Pyridine Hemochromogen Assay and Determination of Extinction Coefficients and Heme Occupancy

Reducing reagents were removed from the purified NC1 and HBD proteins via buffer exchange to avoid interference with the BCA assay. Same protein stock solutions were then used in both pyridine hemochromogen and BCA assays. Pyridine hemochromogen assays were performed as described [25]. Briefly, 500 µL of a stock solution containing 0.2 M NaOH and 40% pyridine and 3 µL of 0.1 M potassium ferricyanide were mixed with 500 µL of protein solution in a cuvette. The oxidized absorption spectrum was recorded between 500 and 600 nm using a Cary 300 spectrophotometer (Varian, Palo Alto, CA), with the spectral bandwidth set to 0.5 nm and in single beam mode. A few crystals of sodium dithionite were added to the solution, mixed well, and the reduced spectrum was recorded. Heme concentration was determined using Beer’s law (*A* = *εC*). The *A*_557_ was the absorbance at 557 nm from the reduced spectrum, and the *ε*_557_ is 34.53 mM^−1^cm^−1^. After the heme concentration (*C*) was determined, the extinction coefficients (*ε*) of the DGCR8-Fe(III) heme complex were calculated, again using Beer’s law, from the electronic absorption spectra of the native protein at the wavelengths where only heme absorbed.

Protein concentrations were determined using the Micro BCA Protein Assay kit (Thermo Fisher Scientific, Rockford, IL) following to the manufacturer’s protocol. Heme occupancy is defined as the molar ratio of heme and dimeric proteins in the complexes.

### Crystallization

In an effort to crystallize the frog HBD in a thiol-oxidized state, this protein was treated with 10 mM oxidized glutathione (GSSG) at pH 8.0 for 20 min. GSSG was removed via buffer exchange into a low-salt buffer containing 200 mM NaCl, 20 mM Tris pH 8.0, and 5 mM DTT. Crystallization trials were set up using a Mosquito robot (TTP LabTech, Cambridge, MA) and hanging drop vapor diffusion method. Frog HBD at ∼10 mg/mL was mixed in a 1∶1 (v/v) ratio with a well solution containing 20% (w/v) polyethylene glycol (PEG)-1000, 0.1 M imidazole pH 8.0, and 0.2 M calcium acetate, with 1 M tribasic sodium citrate added to the drop to 10% of final drop volume. Crystals were grown at 18°C in 4 months. Single crystals were soaked in a cryo-protection solution, containing all the components of the well solution and 20% glycerol, for 5 min at 18°C, mounted with CrystalCap HT Cryoloops (Hampton Research), and flash-frozen in liquid nitrogen prior to data collection.

### Data Collection, Structure Determination, Refinement and Analysis

Diffraction images were collected using a Rigaku R-AXIS-IV++ imaging plate detector and Cu Kα X-ray radiation from a Rigaku FRE+ rotating anode generator with confocal optics. Data were processed using HKL2000 [32]. Initial phases were calculated via molecular replacement using the program PHASER [33], [34] with the human dimerization domain structure (PDB code 3LE4) as the search model. An initial model was built using ARP/wARP [35], followed by manual model building using COOT [36] and refinement using REFMAC [37] and PHENIX [38]. Structural superposition was performed using the program MAPS [39]. Coordinates and structure factor amplitudes have been deposited in the RCSB Protein Data Bank with accession code 4E5R.

### Mass Spectrometry

Crystals were dissolved in a buffer containing 400 mM NaCl, 20 mM Tris pH 8.0, and 1 mM DTT. The solution was desalted using Omix C18 tips (Agilent Technologies, Santa Clara, CA) according to the manufacturer’s protocol. Sinapinic acid dissolved in 70% acetonitrile and 0.1% trifluoroacetic acid was used as the matrix and was mixed with the desalted protein solution in a 1∶1 (v/v) ratio. Data were obtained on a Voyager DE STR MALDI-TOF mass spectrometer (Applied Biosystems, Foster City, CA), set in the linear mode and with *m/z* range of 2,000–30,000. Data were plotted using Prism (version 4.0, GraphPad, La Jolla, CA).
